# Correlation Between the COVID-19 Respiratory Triage Score and SARS-COV-2 PCR Test

**DOI:** 10.3389/fmed.2020.605689

**Published:** 2020-12-07

**Authors:** Ahmad Aldobyany, Abdelfattah Touman, Nabil Ghaleb, Rajaa Alsaggaf, Noureen Murtaza, Adel Hamada, Moataz Alknawy, Amr S. Albanna, Eid Alqurashi

**Affiliations:** ^1^Medicine Department, King Abdullah Medical City, Mecca, Saudi Arabia; ^2^King Saud bin Abdulaziz University for Health Sciences, King Abdullah International Medical Research Center, Jeddah, Saudi Arabia; ^3^McGill University, Montreal, QC, Canada

**Keywords:** COVID-19, SARS-CoV-2, respiratory triage, screening for COVID-19, viral pneumonia

## Abstract

**Background:** COVID-19 clinical presentation is usually non-specific and includes commonly encountered symptoms like fever, cough, nausea, and vomiting. It has been reported that COVID-19 patients can potentially transmit the disease to others before developing symptoms. Thus, extensive surveillance and screening of individuals at risk of the disease is required to limit SARS-COV-2 spread. The COVID-19 respiratory triage score has been used for patient screening. We aimed to determine its diagnostic performance characteristics, which have not been adequately studied before.

**Methodology:** This is a retrospective observational study involving all patients screened for COVID-19 at a tertiary care facility. Patients were tested using nasopharyngeal swab for SARS-COV-2 PCR. The Saudi CDC COVID-19 respiratory triage score was measured for all subjects. The sensitivity, specificity, positive predictive value, and negative predicted value of COVID-19 respiratory triage score were measured with reference to SARS-COV-2 PCR test. Multivariate regression analysis was done to identify factors that can predict a positive SARS-COV-2 PCR test.

**Result:** A total of 1,435 subjects were included. The COVID-19 respiratory triage score provided a marginal diagnostic performance with a receiver-operating characteristics (ROC) area under the curve value of 0.60 (95% CI: 0.57–0.64). A triage score of 5 provided the best cut-off value for the combined sensitivity and specificity. Clinical characteristics that independently predicted positive COVID-19 PCR test include male sex (adjusted OR: 1.47; *p* = 0.034), healthcare workers and their family members (adjusted OR: 1.99; 95%; *p* = 0.016), fever (adjusted OR: 2.98; *p* < 0.001), and moderate disease severity (adjusted OR: 5; *p* < 0.001).

**Conclusion:** The current COVID-19 respiratory triage score has marginal diagnostic performance characteristics. Its performance can improve by including additional predictors to the respiratory symptoms in order to avoid missing COVID-19 patients with atypical presentation and to limit unnecessary SARS-COV-2 PCR testing.

## Introduction

In December 2019, a novel coronavirus infection appeared in Wuhan, China, the disease termed corona virus disease 2019 (COVID-19) (1). Over a short duration, the disease rapidly spread globally and was declared a pandemic by the World Health Organization (WHO) in March 2020 (2).

To mitigate the spread of the virus, several measures have been implemented by healthcare organizations and governmental agencies, including social distancing, isolation, and home confinement. But these measures can result in psychosocial strain, lower life satisfaction, and unhealthy eating behaviors (3, 4). Home confinement had a negative impact on mental wellbeing and emotional status with more individuals developing depression and seeking psychosocial support (5). It has been demonstrated that maintenance of home-based Physical Activity programs, especially in vulnerable individuals, is a useful strategy to mitigate the psychosocial strain and disease severity during home confinement (6). Based on multiple studies, there are different activities to overcome these negative effects, including exergaming, participation in yoga, and 150 min of moderate-intensity activity divided in to 5–7 sessions per week (7).

COVID-19 clinical presentation is non-specific with estimated proportions of 44% for fever, 68% for cough, and 5% for nausea and vomiting (8). It has been reported that asymptomatic carriers can potentially transmit COVID-19 to others before developing symptoms (9). Thus, extensive surveillance and screening of individuals at risk is required to limit SARS-COV-2 spread.

The WHO and the Saudi Center for Disease Prevention and Control have published a case suspect definition (10, 11). Both organizations encourage healthcare workers to practice a high level of clinical suspicion and adopt a respiratory triage score system. The respiratory triage is composed of multiple items that include both exposure risk and clinical related parameters (Table 1). The Saudi CDC triage score (version-3) recommended a score of 4 and more as the cut off for isolation and testing (11).

**Table 1:** COVID-19 respiratory triage score.

**Risk for acute respiratory illnesses**	**Score**	
**(A) Exposure risks**	**Adult/Pediatric**	
A history of travel abroad during the 14 days prior to symptoms onset. OR Visiting or being a resident of a high-risk area for COVID-19 in the kingdom during the 14 days prior to symptom onset. (Holy City of Makkah, Madinah City, Riyadh City, Jeddah City, AlHafuf City, AlQatif City). OR A close physical contact with a confirmed case of COVID-19 or MERS-COV in the past 14 days. OR An exposure to camel or camel products in the past 14 days. OR Working in a healthcare facility.	/3	
**(B) Clinical signs, symptoms, and medical history**	**Pediatric**	**Adult**
1. Fever or recent history of fever.	/1	/2
2. Cough (new or worsening).	/1	/2
3. Shortness of breath (new or worsening).	/1	/2
4. Nausea, vomiting, and/or diarrhea.	–	/1
5. Chronic renal failure, CAD/heart failure, Immunocompromised patient.	–	/1
6. Total score	/6	/11

*A score ≥ 4, ask the patient to perform hand hygiene, wear a surgical mask*.

*Follow isolation precaution and then contact The Operation Department for further instructions*.

We have limited data on the sensitivity and specificity of the adopted respiratory triage score. It has not been shown in previous clinical studies whether or not a respiratory triage score correlates to a positive PCR test for COVID-19. In this study, we aimed to answer the following questions: What is the sensitivity, specificity, positive predictive value, and negative predicted value of this score to the diagnosis of COVID-19? Which factor (fever, cough, SOB, GI symptoms, comorbidities, disease severity, exposure risk, self-presentation, and being a healthcare worker) can predict positive SARS-COV-2 PCR?

## Methods

This is a retrospective observational study involving all patients screened for COVID-19 at King Abdullah Medical City (KAMC). KAMC is a tertiary specialized medical city, composed of 550 beds and located in Makkah, Saudi Arabia. We included all subjects presented to KAMC and tested for COVID-19, using a nasopharyngeal swab for SARS-COV-2 PCR, whether they were symptomatic (patient) or screened as contacts (healthy). We excluded subjects who did not have a documented COVID-19 respiratory triage score and those screened using earlier score versions (i.e., all cases presented earlier to 2nd April 2020). RealStar® SARS-CoV-2 RT-PCR Kit is a real-time RT-PCR test intended for the qualitative detection of nucleic acid from SARS-CoV-2 was used in authorized laboratories.

All SARS-COV-2 test results were collected from the molecular department where all records are stored. The Saudi CDC COVID-19 respiratory triage score was identified from patients' records (Table 1). Subjects' demographic and clinical data were collected from the electronic file of the patients. Disease severity was classified as the following: mild for flu like or upper respiratory tract symptoms (e.g., fever, cough, sore throat, anosmia, ageusia, mild gastrointestinal symptoms, and headache; moderate for any form of Pneumonia with mild symptoms; and severe if the respiratory rate is ≥30/min, blood oxygen saturation is ≤93%3, PaO2/FiO2 ratio is <300, or there is radiological lung infiltrate of more than 50% of the lung field.

### Statistical Analysis

All analyses were performed using STATA (StataCorp. 2011. Stata Statistical Software: Release 12. College Station, TX: StataCorp LP) software. The proportion and mean for categorical and continuous variables, respectively, were measured to describe patients' characteristics. Using a COVID-19 PCR test as a reference value, the receiver-operating characteristics (ROC) curve and the sensitivity and specificity of different values of COVID-19 triage score were measured. We considered the following cut-off values for interpretation of the area under the ROC curve (AUC): 0.5 to 0.6 has poor, 0.6 to 0.7 has marginal, 0.7 to 0.8 has acceptable, 0.8 to 0.9 has excellent, and more than 0.9 has outstanding discrimination (i.e., ability to diagnose patients with and without the disease) (12). Clinical characteristics that predict positive COVID-19 test result were determined using multiple logistic regression analysis. Statistical significance was determined using the 95% confidence interval and *p*-value of 0.05.

Ethical consideration: Ethical approval from KAMC Institutional Review Board was taken (approval number: 20-636). The informed consent was waived considering the retrospective nature of the study.

## Result

A total of 1,435 subjects were included in the study. The baseline demographic and disease characteristics are shown in Table 2.

**Table 2:** Baseline patients' demographic and disease characteristics.

**Characteristics**	**COVID-19 (no. 340)**		**Control (-ve for COVID) (no. 1095)**		***P-v*alue**
	**Estimate**	**95% CI**	**Estimate**	**95% CI**	
Age (mean, years)	38.7	37.2–40.2	39.3	38.3–40.2	0.543
Male sex (%)	68.5	63.3–73.4	55.4	52.4–58.4	<0.001
Exposure risk (%)	96.5	93.9–98.2	98.5	97.5–99.1	0.024
**Reason for COVID-19 test (%)**					
Active Screening*	30.0	25.2–35.3	50.1	47.1–53.2	<0.001
Self-Presentation	70.0	64.7–74.8	49.9	46.8–52.9	
**Relation to Health Facility (%)**					
Health Care Worker (HKW)	74.9	68.6–80.5	69.2	65.7–72.6	0.015
Relative to HKW	5.0	2.5–8.8	2.5	1.5–3.9	
None	20.1	15.0–26.0	28.3	25.0–31.7	
**Comorbidities (%)**	10.9	7.8–14.7	19.3	17.0–21.8	<0.001
**Symptoms (%)**					
Asymptomatic	23.3	18.8–28.4	44.7	41.6–47.8	<0.001
Fever	50.6	45.1–56.0	25.4	22.9–28.1	<0.001
Cough	42.1	36.8–47.5	23.2	20.7–25.8	<0.001
Dyspnea	15.0	11.4–19.2	7.7	6.2–9.4	0.466
GI symptoms	7.6	5.1–11.0	7.7	6.2–9.4	0.985

*COVID-19, Coronavirus disease 2019; GI, gastrointestinal*.

* *Active screening of contacts to COVID-19 patients or recently traveled subjects*.

COVID-19 triage score had marginal performance characteristics, compared to the PCR test as a reference standard, with best combination of sensitivity and specificity values at score 5 (better specificity, without significant reduction in sensitivity, than the current cut-off score 4).

**Figure 1:**
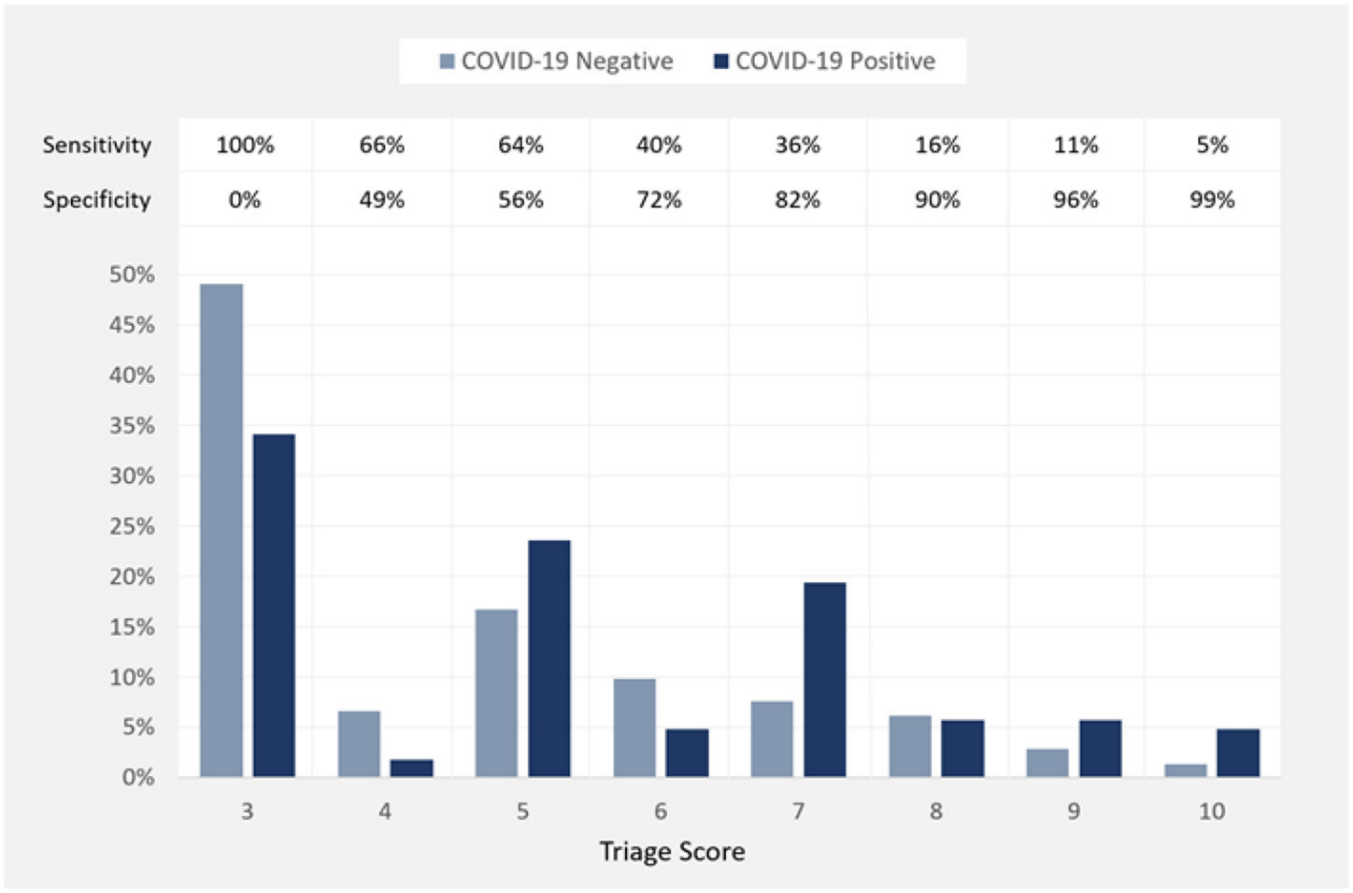
Percentage of negative and positive COVID-19 PCR test results at each value of COVID-19 Triage Score and the performance characteristics (sensitivity and specificity) of these values.

Score 4 sensitivity, specificity, positive predictive value, and negative predictive value are 65.9, 49.1, 28.8, and 82.1%, respectively. Score 5 sensitivity, specificity, positive predictive value, and negative predictive value are 64, 55.7, 31.1, and 83.2%, respectively (Figure 1 and Table 3).

**Table 3:** Performance characteristics of COVID-19 Triage Score at Cut-off Values of 4 and 5.

	**Percentage**	**95% CI**
**COVID-19 triage score cut-off value of 4**		
Sensitivity	65.9	60.5–71.0
Specificity	49.1	46.0–52.1
PPV	28.8	25.6–32.1
NPV	82.1	78.9–85.1
**COVID-19 triage score cut-off value of 5**		
Sensitivity	64.0	58.6–69.2
Specificity	55.7	52.6–58.7
PPV	31.1	27.6–34.7
NPV	83.2	80.3–85.9

*COVID-19, Coronavirus disease 2019; PPV, Positive predictive value; NPV, Negative predictive value*.

**Figure 2:**
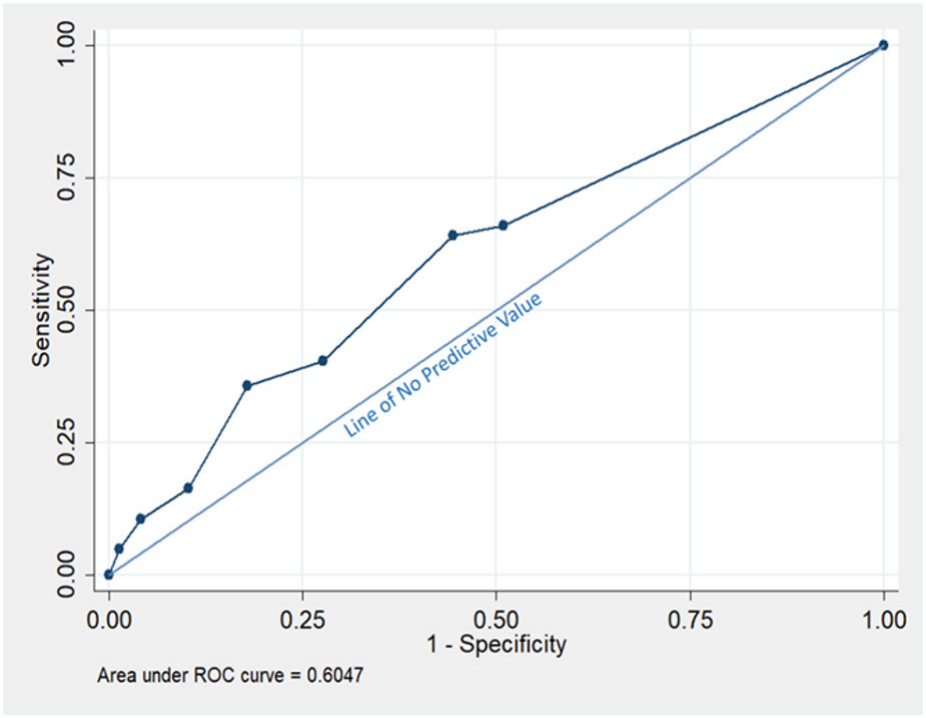
The Receiver operating characteristic (ROC) of COVID-19 Triage Score using the COVID-19 PCR testing as a Reference Value.

The receiver operating characteristics ROC of COVID-19 respiratory triage score was above the line of no predictive value with an area under the curve AUC value of 0.60 (95% CI: 0.57–0.64) (Figure 2), indicating satisfactory performance to predict positive PCR test result.

Clinical characteristics that independently predict positive COVID-19 PCR test include male sex (adjusted OR: 1.47; 95% CI: 1.03–2.09; *p* = 0.034), healthcare workers and their family members (adjusted OR: 1.99; 95% CI: 1.14–3.5; *p* = 0.016), fever (adjusted OR: 2.98; 95% CI: 1.97–4.5; *p* < 0.001), and moderate disease severity (adjusted OR: 5; 95% CI: 1.23–20; *p* < 0.001) (Table 4).

**Table 4:** Clinical characteristics that predict positive COVID-19 PCR test among screened patients.

**Characteristics**	**Crude OR**	**95% CI**	**P-value**	**Adjusted OR**	**95% CI**	**P-value**
Age (years)	0.997	0.989–1.00	0.543	1.004	0.99–1.03	0.649
Male sex	**1.75**	**1.35**–**2.27**	**<0.001**	**1.47**	**1.03**–**2.09**	**0.034**
Exposure risk	**0.43**	**0.20**–**0.91**	**0.028**	1.03	0.14–7.68	0.980
Self-presentation*	**2.34**	**1.80**–**3.05**	**<0.001**	1.66	0.82–3.39	0.162
Health care worker**	**1.57**	**1.08**–**2.27**	**0.017**	**1.99**	**1.14**–**3.50**	**0.016**
Comorbidities	**0.51**	**0.35**–**0.74**	**<0.001**	0.60	0.31–1.13	0.112
Fever	**3.01**	**2.33**–**3.87**	**<0.001**	**2.98**	**1.97**–**4.50**	**<0.001**
Cough	**2.40**	**1.86**–**3.10**	**<0.001**	1.37	0.91–2.07	0.131
Dyspnea	1.14	0.81–1.60	0.466	0.83	0.48–1.46	0.521
GI symptoms	0.995	0.63–1.57	0.985	0.76	0.42–1.38	0.374
**Severity of symptoms**						
Asymptomatic	Ref	Ref	Ref		Ref	Ref
Mild	**2.57**	**1.92**–**3.45**	**<0.001**	0.96	0.45–2.04	0.914
Moderate	**3.89**	**1.95**–**7.75**	**<0.001**	**5.00**	**1.23**–**20.4**	**0.025**
Severe	**4.14**	**1.64**–**10.5**	**0.003**	0.97	0.14–6.89	0.973
Critical	**2.39**	**1.11**–**5.16**	**0.026**	1.71	0.50–5.86	0.391

*OR, odds ratio; GI, gastrointestinal*.

* *Patient presented to emergency or clinic as opposed to active screening of contacts or recent travelers*.

** *Including both health care workers and their family members. Values with significant P value were bold*.

## Discussion

Early diagnosis and isolation of COVID-19 patients are important to limit the SARS-COV-2 spread and start treatment early in the course of the disease. In this research, we compared the performance of the COVID-19 respiratory triage score with reference to SARS-COV-2 PCR testing and found the best combination of sensitivity and specificity values at score 5, 64%, and 56%, respectively. The ROC of the score was above the line of no predictive value with an AUC value of 60%. Clinical characteristics that independently predict positive COVID-19 PCR test include male sex, health care workers and their family members, fever, and moderate disease severity.

Universal screening using SARS-COV-2 PCR testing is the most sensitive way to identify and diagnose patients with COVID-19; however, it might not be cost-effective especially in countries with low disease prevalence or limited resources. In New-York-Presbyterian Allen Hospital and Columbia University Irving Medical Center, all pregnant women admitted for delivery were screened using nasopharyngeal SARS-COV-2 PCR testing. Out of 211 women, 29 (13.7%) tested positive (13). In Saudi Arabia, where the community transmission of COVID-19 is much lower compared to New York, universal PCR testing may not be cost-effective due to lower estimated rate of positive COVID-19 results.

Based on US CDC guidelines, triage process is done based on the presence or absence of one of respiratory symptoms (fever, cough, or shortness of breath). Then infection control program will be contacted after patient evaluation and separation in a single room (14). We found that 23% of positive COVID-19 patients were asymptomatic. Those asymptomatic individuals carry high risk for disease transmission if unrecognized through triaging and testing. We demonstrated in this research that factors other than respiratory symptoms can strongly predict COVID-19 like male sex and being healthcare worker or their family members.

Depending on respiratory symptoms or fever alone to screen for COVID-19 might lead to underdiagnosis of the disease. In one study, fever was present in only 43.8% at the time of admission; however, fever prevalence increased to 89% during hospitalization (15). This warrants the use of well structure COVID-19 triage score to detect COVID-19 patient early during the course of the disease even before symptoms appear.

In our study we found that fever (50.6%) is the most frequently reported symptom. This is followed by cough (42.1%), shortness of breath (15%), and then gastrointestinal symptoms (7.6%). The prevalence of symptoms in our study is lower but follow the same trend compared to what has been published in a systematic review showing higher rates of fever (83.3%), cough (60.3%), and fatigue (38.0%), followed by increased sputum production, shortness of breath, and myalgia (16). Other studies report variation of the rate of fever ranging from 83 to 98% at the time of admission (1, 17). Extensive screening of our population may explain the lower rate of fever, compared to published studies.

It is important to acknowledge the limitations of our study, which include the retrospective design and the selective screening of symptomatic or contacts rather than universal screening of all patients presenting to KAMC. On the other hand, we included a large number of subjects, followed a standard protocol implemented by well-trained COVID team members, and conducted the study in a single center to avoid protocol deviation.

Our study findings have important clinical implications as they indicate that the diagnostic performance of the currently utilized triage scoring requires further improvement. This can be achieved by implementing additional disease predictors, which are significantly associated with positive PCR testing.

## Conclusion

The currently utilized triage scoring system for identifying COVID 19 patients has marginal diagnostic value with a best sensitivity and specificity combination at the score of 5 rather than the currently used cut-off score of 4. We also identified additional important predictors that can be integrated to the triage scoring system to improve its diagnostic performance.

## Data Availability Statement

The original contributions presented in the study are included in the article/supplementary materials, further inquiries can be directed to the corresponding author/s.

## Ethics Statement

The studies involving human participants were reviewed and approved by King Abdullah Medical City Institutional Review Board. Written informed consent from the participants' legal guardian/next of kin was not required to participate in this study in accordance with the national legislation and the institutional requirements.

## Author Contributions

AA was the principal investigator. AT, NG, RA, NM, and AH performed the data entry and reviewed the manuscript. ASA performed the statistics analysis and reviewed the manuscript. EA and MA reviewed the manuscript. All authors contributed to the article and approved the submitted version.

## Conflict of Interest

The authors declare that the research was conducted in the absence of any commercial or financial relationships that could be construed as a potential conflict of interest.

## References

[1] Huang C Wang Y Li X Ren L Zhao J Hu Y. Clinical features of patients infected with 2019 novel coronavirus in Wuhan, China. Lancet. (2020) 395:497–506. 10.1016/S0140-6736(20)30183-5 31986264 PMC7159299

[2] Archived: WHO Timeline - COVID-19 Available online at: https://www.who.int/news-room/detail/27-04-2020-who-timeline—covid-19 (Retrieved August 15, 2020).

[3] Ammar A Chtourou H Boukhris O Trabelsi K Masmoudi L Brach M. COVID-19 home confinement negatively impacts social participation and life satisfaction: a worldwide multicenter study. Int J Environ Res Public Health. (2020) 17:6237. 10.3390/ijerph17176237 32867287 PMC7503681

[4] Ammar A Brach M Trabelsi K Chtourou H Boukhris O Masmoudi L. Effects of COVID-19 home confinement on eating behaviour and physical activity: results of the ECLB-COVID19 international online survey. Nutrients. (2020) 12:1583. 10.3390/nu12061583 32481594 PMC7352706

[5] Ammar A Trabelsi K Brach M Chtourou H Boukhris O Masmoudi L Effects of home confinement on mental health and lifestyle behaviours during the COVID-19 outbreak: insight from the ECLB-COVID19 multicenter study. Biol Sport. (2020) 38:9–21. 10.5114/biolsport.2020.96857 PMC7996377 33795912

[6] Bentlage E Ammar A How D Ahmed M Trabelsi K Chtourou H. Practical recommendations for maintaining active lifestyle during the COVID-19 pandemic: a systematic literature review. Int J Environ Res Public Health. (2020) 17:6265. 10.3390/ijerph17176265 32872154 PMC7503956

[7] Chtourou H Trabelsi K H'mida C Boukhris O Glenn JM Brach M. Staying physically active during the quarantine and self-isolation period for controlling and mitigating the COVID-19 pandemic: a systematic overview of the literature. Front Psychol. (2020) 11:1708. 10.3389/fpsyg.2020.01708 33013497 PMC7466737

[8] Guan WJ Ni ZY Hu Y Liang WH Ou CQ He JX Clinical characteristics of coronavirus disease 2019 in China. N Engl J Med. (2020) 382:1708–20. 10.1056/NEJMoa2002032 32109013 PMC7092819

[9] Bai Y Yao L Wei T Tian F Jin DY Chen L. Presumed asymptomatic carrier transmission of COVID-19. JAMA. (2020) 323:1406–7. 10.1001/jama.2020.2565 32083643 PMC7042844

[10] Global Surveillance for Human Infection With Coronavirus Disease (COVID-19) Available online at: https://www.who.int/publications/i/item/global-surveillance-for-human-infection-with-novel-coronavirus-(2019-ncov) (Retrieved August 15, 2020).

[11] Professionals and Health Workers (2020). Available online at: https://covid19.cdc.gov.sa/wp-content/uploads/2020/04/AREN_Quick-Guide-to-COVID-19-Surveillance-Case-Definitions-and-Disposition-v2.pdf (Retrieved August 15, 2020).

[12] Mandrekar JN. Receiver operating characteristic curve in diagnostic test assessment. J Thorac Oncol. (2010) 5:1315–6. 10.1097/JTO.0b013e3181ec173d 20736804

[13] Sutton D Fuchs K D'Alton M Goffman D. Universal screening for SARS-CoV-2 in women admitted for delivery. N Engl J Med. (2020) 382:2163–4. 10.1056/NEJMc2009316 32283004 PMC7175422

[14] Standard Operating Procedure (SOP) for Triage of Suspected COVID-19 Patients in Non-US Healthcare Settings: Early Identification and Prevention of Transmission during Triage Available online at: https://www.cdc.gov/coronavirus/2019-ncov/hcp/non-us-settings/sop-triage-prevent-transmission.html (Retrieved August 15, 2020).

[15] Guan WJ Ni ZY Hu Y Liang WH Ou CQ He JX Clinical characteristics of coronavirus disease 2019 in China. N Engl J Med. (2020) 382:1708–20. 10.1101/2020.02.06.20020974 32109013 PMC7092819

[16] Fu L Wang B Yuan T Chen X Ao Y Fitzpatrick T. Clinical characteristics of coronavirus disease 2019 (COVID-19) in China: a systematic review and meta-analysis. J Infect. (2020) 80:656–65. 10.1016/j.jinf.2020.03.041 32283155 PMC7151416

[17] Chen N Zhou M Dong X Qu J Gong F Han Y. Epidemiological and clinical characteristics of 99 cases of 2019 novel coronavirus pneumonia in Wuhan, China: a descriptive study. Lancet. (2020) 395:507–13. 10.1016/S0140-6736(20)30211-7 32007143 PMC7135076

